# Urine Discoloration Associated with Metronidazole: A Case Report

**DOI:** 10.31729/jnma.7889

**Published:** 2022-11-30

**Authors:** Mohammad Firoz Anjum, Sajal Twanabasu, Kipa Shrestha

**Affiliations:** 1 Department of Paediatrics, Patan Academy of Health Sciences, Lagankhel, Lalitpur, Nepal; 2 Department of General Practice and Emergency Medicine, Patan Academy of Health Sciences, Lagankhel, Lalitpur, Nepal

**Keywords:** *flagyl*, *hematuria*, *metronidazole*, *side effects of drugs*

## Abstract

Metronidazole belongs to the nitroimidazole group of antibiotics. It is used for the treatment of anaerobic and protozoal organisms. Its common side effects are nausea, vomiting, headache, and abdominal pain. Urine discolouration is one of the rare side effects of metronidazole. We report a case of 4-years-old male, a known case of Steroid resistant nephrotic syndrome; Focal Segmental Glomerulosclerosis type who developed reddish discolouration of urine following the introduction of oral metronidazole which reverted back to its usual colour within 24 hours of discontinuing metronidazole. With no evidence of other possible causes, urine discolouration was linked with the use of metronidazole. Urine discoloration as a side effect of metronidazole is rarely reported in the literature. This case report of the rare adverse effects of the commonly used drug can be of importance in the scientific community. Urine discolouration could be considered a side effect of metronidazole when there is no other obvious cause.

## INTRODUCTION

Metronidazole is a nitroimidazole antibiotic, which is bactericidal against gram-positive and gramnegative anaerobic bacteria.^1^ Common adverse effects of metronidazole include gastrointestinal upset, headache, and taste disturbances.^2^ There are only a few case reports regarding urine discolouration associated with metronidazole use.^3,4^ The Physician's Desk Reference showed dark urine as a side effect of metronidazole in 1 in 100,000 users of the medication.^5^ Here we report a case of a patient who developed reddish discolouration of urine following the introduction of oral metronidazole.

## CASE REPORT

A 4-years-old male child was admitted to the children's ward of a tertiary care centre with the diagnosis of Steroid Resistant Nephrotic Syndrome; Focal Segmental Glomerulosclerosis (FSGS) type who was under mycophenolate mofetil (35 mg/kg/day in 2 divided doses), tacrolimus (0.15 mg/kg/day once a day), prednisolone (60 mg/m^2^/day), amlodipine (0.2 mg/kg/day once a day) and enalapril (0.3 mg/kg/day) developed multiple episodes of loose stool and vomiting. On examination, some signs of dehydration were present with stable vitals and systemic examination was unremarkable.

**Figure 1:**
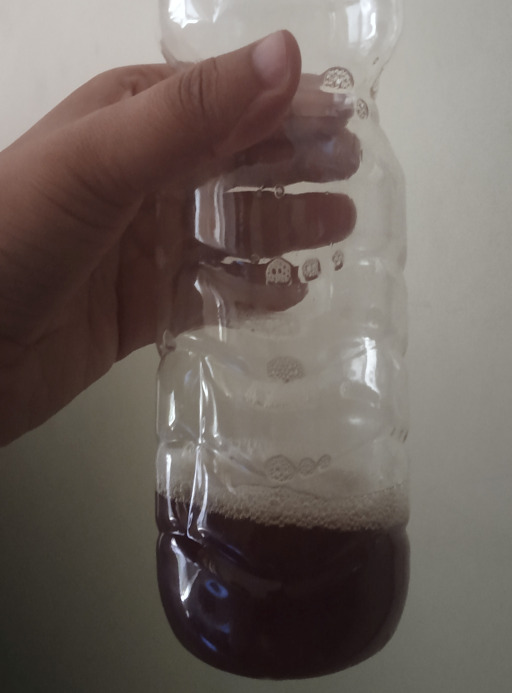
Urine colour after initial administration of metronidazole.

Routine and microscopic stool examination was normal. The child was started on zinc tablets (20 mg once a day) and Oral Rehydration Solution (ORS). Urine routine and microscopic examination showed pus cells of 10-15/high power field (HPF). A tablet of ofloxacin (15 mg/kg/day) was started for pyuria. Since loose stool still persisted, mycophenolate mofetil was put on hold as it can also cause diarrhoea. However, loose stool still continued, oral metronidazole (30 mg/kg/day) was started empirically and ofloxacin was discontinued. Two days after starting metronidazole, there was the passage of dark red-coloured urine (Figure 1).

A urine microscopic examination was done which was negative for hematuria. Urine for haemoglobin was also negative. There was no past history of urine discolouration with his prior usual medications. Moreover, no new drug except metronidazole was introduced. Hence, a potential cause of reddish discolouration of urine due to adverse effects of other drugs was ruled out.

**Figure 2:**
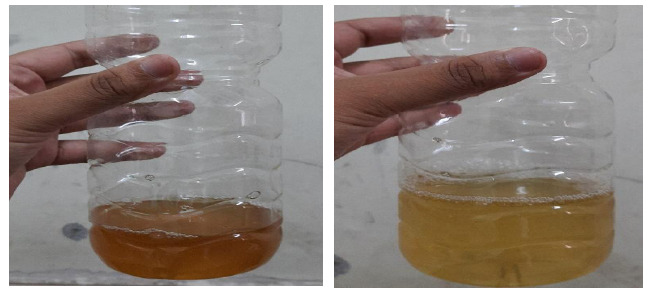
Subsequent urine colour after discontinuation of metronidazole.

Furthermore, there was no evidence suggestive of hemolysis or rhabdomyolysis leading to urine discolouration. The creatinine phosphokinase (CPK) testing was not done as there was no history suggestive of rhabdomyolysis. Similarly, there were no symptoms pertaining to ongoing hemolysis like an increase in pallor or yellowish discolouration of the eyes; objective testing for hemolysis was not done. Also, there was no history of intake of any foods such as beets, blackberry, or rhubarb which could also cause dark red-coloured urine. Urine colour returned to its usual colour within 24 hours of the termination of metronidazole (Figure 2).

Metronidazole was not reintroduced as discoloration of urine was visually upsetting to the patients and the treating physician. The child improved symptomatically.

## DISCUSSION

Urine analysis is important in the evaluation of illness and the colour of urine forms part of this assessment. Abnormal urine colour is a known side effect of a number of drugs. Rifampicin can cause orange-coloured urine, vitamin B complex causes deep yellow colour urine, and phenytoin and nitrofurantoin can cause brown colour urine.^6^ Metronidazole is only rarely mentioned for urine discolouration. A review of Goldfrank's Toxicology showed no evidence of this side effect.^7^

It has been hypothesised that the pigment of an azo metabolite is responsible for metronidazole-induced urine discolouration.^8^ The metabolite leads to a change in urine colour but it doesnot have any clinical significance. Food such as beets, blackberries, and rhubarb can cause red-coloured urine.^9^ Focal segmental glomerulosclerosis rarely causes red-coloured urine or hematuria.

There are very few case reports showing an association between the use of metronidazole and reddish discolouration of urine. The first case reported was more than 40 years ago.^3^ Published article has reported a link between urine discolouration with metronidazole tablet overdose in 2001.^4^ The authors stated that urine discolouration usually occurred with medication overdose or in patients with hepatic dysfunction. Another article has also reported urine discolouration with the use of metronidazole in a 52-year-old male being treated for Clostridium difficile colitis.^10^

Our patient developed reddish discolouration of urine after the introduction of metronidazole. Urinalysis was normal with negative urine for haemoglobin. There was no evidence suggestive of rhabdomyolysis and hemolysis. There was also no history of intake of any foods that can lead to urine discolouration. Urine colour resolved after withdrawal of oral metronidazole. According to the Naranjo adverse drug reaction probability scale, metronidazole was likely responsible for the change in urine colour (score 7-8). An appropriate dose of metronidazole was used and the patient did not have hepatic dysfunction which was similar to the observation by a case report,^10^ and contrasting with the statement by another published study.^4^

Metronidazole is a commonly used drug. Urine discolouration, though listed as one of the adverse effects of Metronidazole, occurs rarely. The clinician may be unaware of the rare side effect of this commonly used drug. This may lead to unnecessary investigations and evaluations. Urine discolouration might be very concerning for patients, their family members and treating physicians even though it is of no clinical significance. This case report can provide an idea about urine discolouration as the side effect of metronidazole and help doctors reassure patients regarding this side effect.
